# A Rare Presentation of Fibromuscular Dysplasia: Postpartum Vascular Catastrophe and Brief Literature Review

**DOI:** 10.1177/2324709617719917

**Published:** 2017-07-31

**Authors:** Fatima Khan, Ali Raza Ghani, Larami Mackenzie, Ashwin Matthew, Usman Sarwar, Bruce Klugherz

**Affiliations:** 1 Department of Internal Medicine Abington Jefferson, PA, USA; 2 Department of Neurovascular, Abington Jefferson Jefferson Health, PA, USA; 3 Drexel University Hospital, PA, USA; 4 Cardiology Abington Jefferson Jefferson Health, PA, USA

**Keywords:** ST segment elevation myocardial infarction, spontaneous coronary artery dissection, acute coronary syndrome, fibromuscular dysplasia, left anterior descending, electrocardiogram, internal carotid artery

## Abstract

Spontaneous coronary artery dissection is a very rare cause of acute coronary syndromes and can be life threatening given the rarity of the condition. It should be part of differentials in young females presenting with acute coronary syndromes without routine risk factors for coronary artery disease, especially before, during, and after pregnancy. It is closely associated with fibromuscular dysplasia and management can be very challenging at times. We present a case of spontaneous coronary artery dissection presenting with recurrent ST segment elevation myocardial infarction in association with fibromuscular dysplasia.

## Case Presentation

**Figure 1.**
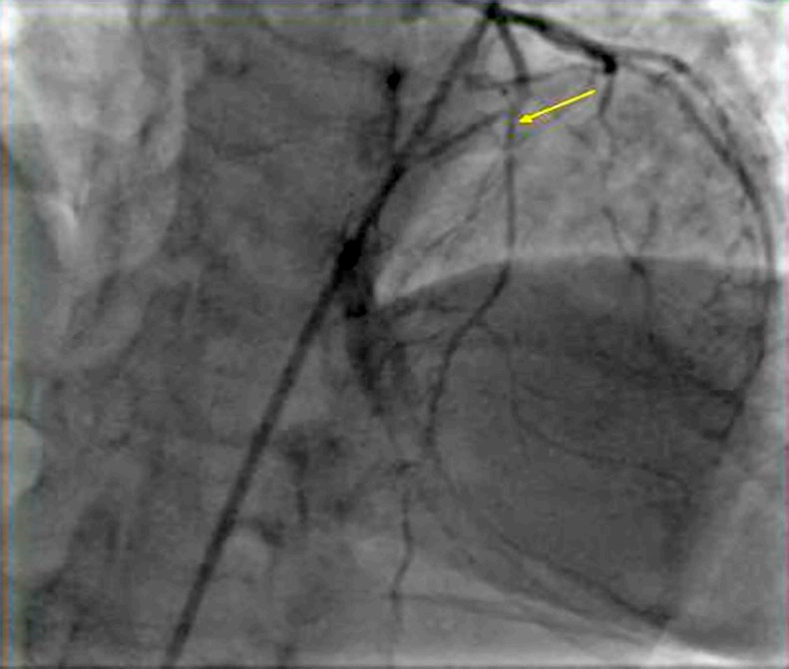
LAD dissection.

**Figure 2.**
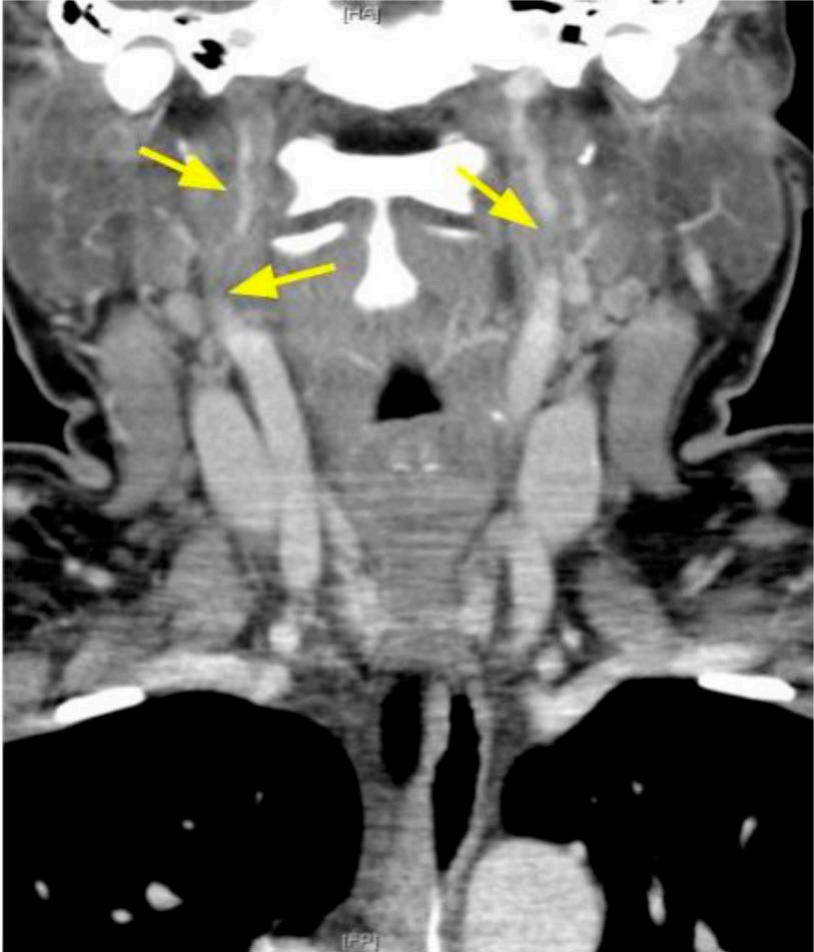
Long segmental stenosis of the right cervical ICA from mid to distal portion. Severe stenosis with luminal narrowing greater than 80%. Mild luminal irregularity of the mid to distal left cervical ICA with 50% stenosis.

A 31-year-old female, 2 weeks postpartum, presented with complaint of chest pain for 3 days. On admission, she was found to have ST segment elevation in leads I and aVL on the electrocardiogram (EKG). The patient was taken to cardiac catheterization laboratory for intervention. Coronary angiography showed spontaneous dissection of the left anterior coronary artery (LAD), and a drug-eluding stent was placed from mid to distal LAD (Figure 1). During the procedure, she had 3 episodes of ventricular tachycardia requiring shock with return of spontaneous circulation. Postprocedure she complained of frontal and occipital headaches. Computed tomography (CT) angiogram of the head and neck showed long segment stenosis of the mid and distal right cervical internal carotid artery (ICA) consistent with dissection. Mild luminal irregularities of the mid to distal left cervical internal carotid artery suggesting 50% stenosis were noted (Figure 2). Since the patient did not complaint of neurologic deficits, no carotid interventions were performed. Given the involvement of multiple vessels, fibromuscular dysplasia (FMD) was suspected and a duplex ultrasound of the renal artery was done, which showed borderline mid right and mid left renal artery peak systolic velocity to aortic velocity ratio, which confirmed the diagnosis of FMD. The patient was sent home on aspirin and ticagrelor with plan to follow-up as an outpatient.

**Figure 3.**
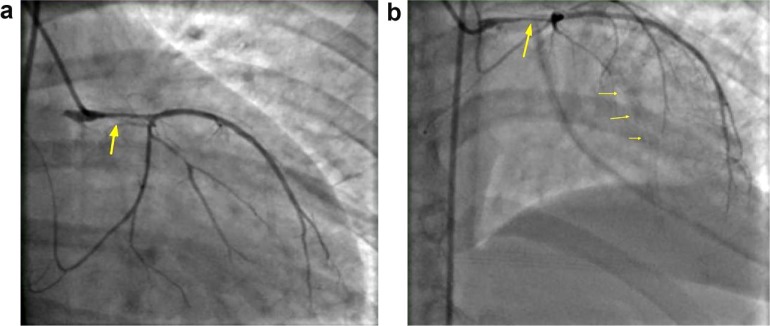
(a) Left main artery dissection. (b) Left main dissection compromising blood flow to LAD.

**Figure 4.**
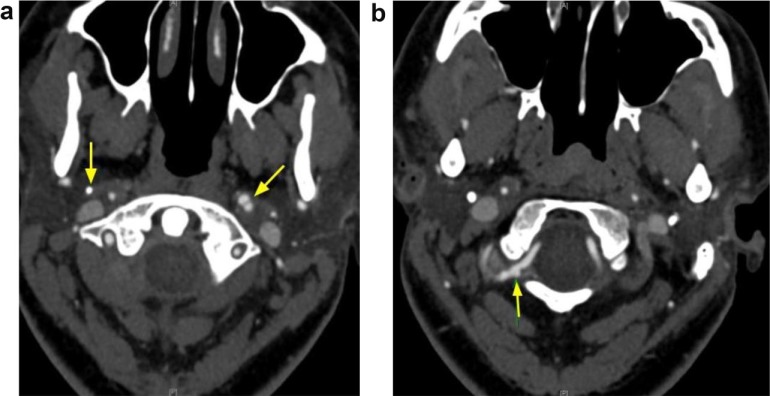
(a) Stenosis of the right cervical ICA and new pseudoaneurysm of the left cervical ICA. (b) Pseudoaneurysm of the right vertebral artery.

The patient returned within 48 hours with complaints of chest pain and worsening headaches. EKG showed ST elevation in leads I, aVL, and V2-V4. Coronary angiography showed dissection of left main artery and left circumflex (Figure 3a and b), which were difficult to stent. The patient eventually underwent coronary artery bypass surgery (CABG). A repeat CT angiogram of the head and neck showed new diffuse narrowing of the left cervical ICA consistent with a dissection and a pseudoaneurysm. A new pseudoaneurysm was noted in the right cervical ICA and the right vertebral artery (Figure 4a and b). No neurologic deficits were present during readmission and intervention was deferred. Unfortunately, the patient developed an apical thrombus in the left ventricle and warfarin was initiated along with aspirin prior to hospital discharge.

## Discussion

Spontaneous coronary artery dissection (SCAD) is classified based on its angiographic appearance.

Type 1 SCAD is an intimal tear of the main vessel or rupture of the vasa vasorum resulting in intramural hematoma with medial dissection, presenting as multiple lumens on angiography.

Type 2 SCAD is the most common type. It spares the intima and appears as diffuse long stenosis on angiography. Type 3 SCAD has an atherosclerotic appearance. Intravascular imaging is mandatory to confirm type 3 SCAD as it cannot be differentiated from atherosclerosis.^1,2^

SCAD is the manifestation of an underlying condition. It is associated with atherosclerotic and several nonatherosclerotic risk factors, such as connective tissue disorders, including FMD, postpartum status, breastfeeding, coronary artery spasm, hormonal therapy, intense emotional stress, and intense exercise.^3,4^

Fifty to 75% of patients with SCAD were found to have coexisting FMD with prominent involvement of the renal, iliac, or cerebrovascular vessels on imaging-based screening. Approximately 14% of such patients were also found to have concomitant intracranial aneurysms.^5^ Pregnancy and FMD are independent risk factors for SCAD. Unfortunately, FMD diagnosis is often delayed for several years in patients who present with hypertension as FMD is poorly understood. Both FMD and pregnancy tend to be associated with multivessel involvement.^6,7^

SCAD involves the mid to distal LAD artery in the majority of cases, and the proximal LAD artery and the left main (LM) coronary in pregnancy-related (p)-SCAD.^8^

Percutaneous coronary intervention (PCI) is the preferred method of intervention in all symptomatic cases of SCAD, except when the LM coronary and ostial LAD artery are involved, in which case coronary artery bypass graft surgery is the treatment of choice. Since dissection, especially pregnancy-associated SCAD, tends to cause the propagation/extension of dissection in nearly half of all patients, PCI is successful in nearly a quarter of patients.^9,10^

Patients who present with acute coronary syndromes should be treated with PCI. Post-PCI patients should be managed medically especially if they have received a stent or have elevated blood pressure. Medication such as aspirin, heparin, beta-blockers, and nitrates can be used for medical management.^11^ An imaging follow-up is a reasonable approach. Nearly all dissection cases have been shown to have healed on repeat imaging at 4 weeks.

In our patient, as discussed above, both coronary and carotid vessels were involved and the patient was managed initially by PCI and later required CABG. She did have carotid involvement but did not require any intervention given the fact that she was asymptomatic. Patients presenting with chest pains should be worked up aggressively, especially before, during, and after pregnancy given the severity and variability of presentation.

## Conclusion

Patients with SCAD usually present with acute coronary syndromes in the female population and diagnosis can be very challenging and misleading. There are no clear-cut guidelines about management but data so far definitely suggest adopting a conservative approach and using aggressive measures like intervention or even CABG if medical management is not successful. If dissection is suspected, glycoprotein IIb/IIa inhibitors and fibrinolytic agents are contraindicated as their use may predispose the patient to intramural hematoma extension.

## References

[1] Vanzetto G Berger-Coz E Barone-Rochette G Prevalence, therapeutic management and medium-term prognosis of spontaneous coronary artery dissection: results from a database of 11,605 patients. Eur J Cardiothorac Surg. 2009;35:250-254. 19046896 10.1016/j.ejcts.2008.10.023

[2] Saw J Coronary angiogram classification of spontaneous coronary artery dissection. Catheter Cardiovasc Interv. 2014;84:1115-1122. 24227590 10.1002/ccd.25293

[3] Prasad M Tweet MS Hayes SN Prevalence of extracoronary vascular abnormalities and fibromuscular dysplasia in patients with spontaneous coronary artery dissection. Am J Cardiol. 2015;115:1672-1677. 25929580 10.1016/j.amjcard.2015.03.011

[4] Saw J Sedlak T Aymong E Spontaneous coronary artery dissection outcomes and association with fibromuscular dysplasia. Can J Cardiol. 2013;29(10):S256.

[5] Elkayam U Jalnapurkar S Kealey A Pregnancy associated myocardial infarction: Contemporary experience in 150 cases between 2005 and 2011. J Am Coll Cardiol. 2012;59(13):E552. 10.1161/CIRCULATIONAHA.113.002054 24753549

[6] Appleby CE Barolet A Ing D Contemporary management of pregnancy-related coronary artery dissection: a single-centre experience and literature review. Exp Clin Cardiol. 2009;14(1):e8-e16. 19492033 PMC2689090

[7] Persu A Giavarini A Touzé E European consensus on the diagnosis and management of fibromuscular dysplasia. J Hypertens. 2014;32:1367-1378. 24842696 10.1097/HJH.0000000000000213

[8] Saw J Ricci D Starovoytov A Fox R Buller CE Spontaneous coronary artery dissection: Prevalence of predisposing conditions including fibromuscular dysplasia in a tertiary center cohort. JACC Cardiovasc Interv. 2013;6:44-52. 23266235 10.1016/j.jcin.2012.08.017

[9] Tweet MS Hayes SN Pitta SR Clinical features, management, and prognosis of spontaneous coronary artery dissection. Circulation. 2012;126:579-588. 22800851 10.1161/CIRCULATIONAHA.112.105718

[10] Win-Hobson J Roule V Dahdouh Z Sabatier R Lognone T Grollier G Spontaneous coronary artery dissection: one entity with several therapeutic options. Cardiovasc Revascularization Med. 2012;13(3):203 e1. 10.1016/j.carrev.2012.02.001 22475868

[11] Bonacchi M Prifti E Giunti G Emergency management of spontaneous coronary artery dissection. J Cardiovasc Surg. 2002;43:189-193. 11887053

